# Severe Sepsis in Severely Malnourished Young Bangladeshi Children with Pneumonia: A Retrospective Case Control Study

**DOI:** 10.1371/journal.pone.0139966

**Published:** 2015-10-06

**Authors:** Mohammod Jobayer Chisti, Mohammed Abdus Salam, Pradip Kumar Bardhan, Abu S. G. Faruque, Abu S. M. S. B. Shahid, K. M. Shahunja, Sumon Kumar Das, Md Iqbal Hossain, Tahmeed Ahmed

**Affiliations:** 1 Centre for Nutrition & Food Security (CNFS), International Centre for Diarhoeal Disease Research, Bangladesh (icddr,b), Dhaka, Bangladesh; 2 Dhaka Hospital, icddr,b, Dhaka, Bangladesh; 3 Research & Clinical Administration and Strategy (RCAS), icddr,b, Dhaka, Bangladesh; Public Health England, UNITED KINGDOM

## Abstract

**Background:**

In developing countries, there is no published report on predicting factors of severe sepsis in severely acute malnourished (SAM) children having pneumonia and impact of fluid resuscitation in such children. Thus, we aimed to identify predicting factors for severe sepsis and assess the outcome of fluid resuscitation of such children.

**Methods:**

In this retrospective case-control study SAM children aged 0–59 months, admitted to the Intensive Care Unit (ICU) of the Dhaka Hospital of the International Centre for Diarrhoeal Disease Research, Bangladesh from April 2011 through July 2012 with history of cough or difficult breathing and radiologic pneumonia, who were assessed for severe sepsis at admission constituted the study population. We compared the pneumonic SAM children with severe sepsis (cases = 50) with those without severe sepsis (controls = 354). Severe sepsis was defined with objective clinical criteria and managed with fluid resuscitation, in addition to antibiotic and other supportive therapy, following the standard hospital guideline, which is very similar to the WHO guideline.

**Results:**

The case-fatality-rate was significantly higher among the cases than the controls (40% vs. 4%; p<0.001). In logistic regression analysis after adjusting for potential confounders, lack of BCG vaccination, drowsiness, abdominal distension, acute kidney injury, and metabolic acidosis at admission remained as independent predicting factors for severe sepsis in pneumonic SAM children (p<0.05 for all comparisons).

**Conclusion and Significance:**

We noted a much higher case fatality among under-five SAM children with pneumonia and severe sepsis who required fluid resuscitation in addition to standard antibiotic and other supportive therapy compared to those without severe sepsis. Independent risk factors and outcome of the management of severe sepsis in our study children highlight the importance for defining optimal fluid resuscitation therapy aiming at reducing the case fatality in such children.

## Introduction

Severe sepsis is associated with very high case-fatality; however, it’s appropriate management remains a challenge both in the developing and developed countries [1–3]. Often this is the terminal event in acutely ill children with infectious diseases including pneumonia [4–7]; the burden of pneumonia and sepsis is the highest in developing countries [8]. Among the estimated 6.3 million worldwide deaths in under-five children 15% were attributable to pneumonia and over half of the deaths were due to sepsis and the bulk of these deaths occur in Asia and Sub-Saharan Africa [8, 9]. The early recognition of sepsis, before its progression to severe sepsis, and simultaneously aggressive antibiotic therapy may reduce deaths [10, 11]. Additionally, early and efficient fluid resuscitation in children with severe sepsis help to reduce deaths [5]. However, early identification and treatment of severe sepsis in pneumonic severely acute malnourished (SAM) children is a clinical challenge. Pneumonic SAM children lack in the clinical signs for severe sepsis [12–14], despite being very sick, and aggressive fluid management in such children may lead to undesirable effects [15], especially heart failure, cardiac arrest, and deaths [16]. Fluid resuscitation in severely malnourished children had always been very intriguing. The World Health Organization (WHO) recommends relatively slower, less aggressive fluid resuscitation in the management of severely malnourished children with severe sepsis aiming to reduce these adverse effects [16]. Only large trial of rapid fluid resuscitation done in children with features of severe sepsis in developing countries (FEAST trial) showed that fluid boluses were harmful, even without malnutrition [17]. Moreover, there is lack of experimental evidence in current guideline in SAM children. The Intensive Care Unit (ICU) of the Dhaka Hospital of the International Centre for Diarrhoeal Diseases Research, Bangladesh (icddr,b) provides care and treatment to a significant number of pneumonic SAM children with severe sepsis each year, following its guidelines [18] which is very similar to the WHO guideline [19], yet death remains high in such populations. There is lack of data on the predicting factors and outcome of the severe sepsis in pneumonic SAM children. We, therefore, performed retrospective chart analysis to identify factors that predict severe sepsis in pneumonic SAM children who received fluid resuscitation compared with pneumonic SAM children without severe sepsis who did not receive fluid resuscitation. We also evaluated the outcome among the two comparison groups.

## Materials and Methods

### Ethics statement

This research study was approved by the institutional review board [named as Research Review Committee (RRC) and the Ethical Review Committee (ERC)] of icddr,b. A written informed consent was obtained from attending parents or caregivers of each of the participating children; children whose parents/ caregivers did not give consent were not included in the study.

### Study design

We performed retrospective chart analysis and followed unmatched case-control design. SAM children of either sex, aged 0–59 months, admitted to the study hospital from April 2011 through July 2012 with history of cough or difficult breathing and radiologic pneumonia, who were assessed for severe sepsis at admission constituted the study population. Pneumonic SAM children who were assessed to have severe sepsis constituted the cases, and those without severe sepsis constituted the controls. Severe sepsis was defined as tachycardia plus hypothermia (≤35.0°C) or hyperthermia (≥38.5°C), or abnormal WBC count plus poor peripheral perfusion (mean arterial pressure ≤50 mm of Hg and/or absent peripheral pulses or capillary refilling time ≥3 seconds) in the absence of clinical dehydration [20]. Abnormal WBC count was defined if WBC count was >11000/cc or, <4000/cc or, band and neutrophil ration ≥ 0.1, or band>10% [21]. During the chart analysis we found that one sample of blood (2.0 ml) was drawn before giving antibiotics and fluid boluses and was sent to the icddr,b microbiology reference laboratory by the attending physician for culture and sensitivity for all the study children on admission following standard aseptic measures irrespective of presence or absence of their severe sepsis. SAM was defined by WHO anthropometry and has been described elsewhere [22]. Pneumonia was defined by WHO recommended radiologic classifications [23].

### Study Setting

The study children were admitted and treated at the Dhaka Hospital of icddr,b and description of this hospital has been provided elsewhere [22].

### Patient management

Both the cases and control children received antibiotics therapy and micronutrients supplementation following hospital guidelines based on local evidence [18]. The cases additionally received fluid resuscitation i.e. 20 ml per kg of body weight per hour of physiological saline (sodium: 154 mMol/L and chloride: 154 mMol/L) or cholera saline (sodium 133 mMol/L, potassium 13 mMol/L, chloride 98 mMol/L and acetate: 48 mMol/L) up to a maximum of 40 ml over 2 hours in the Intensive Care Unit (ICU) of the hospital [18]. Children without severe sepsis (the controls) did not receive any fluid resuscitation. Non-invasive respiratory support was provided using bubble CPAP among the study children who had severe pneumonia and hypoxemia and/or grunting respiration and invasive respiratory support using mechanical ventilation was given who developed respiratory failure [20].

Justification for use of IV physiological saline in severe sepsis: Isotonic fluid is the choice of fluid in managing severe sepsis is isotonic [24]. As physiological saline/normal saline is considered as one of the isotonic fluids, and clinicians in the Dhaka hospital of icddr,b routinely practice infusion of normal saline as the choice of resuscitation fluid in SAM children following our hospital guideline that is based on robust data from Bangladesh published in the Lancet by Ahmed T et al. in 1999 [18]. Although, WHO specifically recommended few resuscitative fluids in SAM children, in spite of the physiological saline, lack in experimental evidence is the main flaw of this recommendation.

### Measurements

Case Report Forms (CRFs) were developed, pretested, and finalized for acquisition of relevant data. Characteristics analyzed included demographic information [age, sex, poor socio-economic status (monthly income of parents/care givers <US$125), non-breastfeeding from neonatal period, lack of BCG vaccination (if there was no scar mark in upper arm within 4 weeks of the test), lack of DPT, OPV, Hib and Hepatitis vaccination, nutritional status (weight for length Z scores), clinical features such as acute watery diarrhea (AWD) and vomiting, reluctance to feed, presence of thrush and lower chest wall in-drawing, drowsiness, hypoxemia [arterial oxygen saturation (SPO_2_) <90% in air [25], abdominal distension, hypoglycemia (RBS <3.0 mmol/L), bacteremia (bacterial isolate from a single blood sample culture), hypokalemia (serum potassium < 3.5 mmol/L), hyperkalemia (serum potassium > 5.5 mmol/L), hyponatremia (serum sodium < 130.0 mmol/L) and hypernatremia (serum sodium > 150.0 mmol/L), hypocalcemia (serum calcium < 2.12 mmol/L), hypomagnesemia (serum Magnisium < 0.7 mmol/L) and acute kidney injury (serum creatinine > 65.0 mmol/L), metabolic acidosis (serum TCO2 < 17.0 mmol/L) at the time of admission, and hospital acquired infection (clinical evidence of new infection after 48 hours of hospitalization) and outcome.

### Sample size calculation

Assuming an exposure rate among controls of 9% with 80% power, a ratio of 1:7 and desired odds ratio of 3.5 the sample size was calculated to be 50 in case of cases and 350 in case of controls.

### Analysis

All data were entered into SPSS for Windows (version 15.0; SPSS Inc, Chicago) and Epi-Info (version 6.0, USD, Stone Mountain, GA). Differences in proportion were compared by the Chi-square test. Student’s t-test was used to compare the means of normally distributed data and Mann-Whitney test was used for comparison of data that were not normally distributed. A probability of less than 0.05 was considered statistically significant. Strength of association was determined by calculating odds ratio (OR) and their 95% confidence intervals (CIs). In identifying risks for severe sepsis in pneumonic SAM children, variables were initially analyzed in a uni-variate model (Table 1), and then risks independently associated with severe sepsis were identified using step-wise logistic regression analysis after controlling for the co-variates (Table 2).

**Table 1:** Admission characteristics of under-five children with severe acute malnutrition with pneumonia and severe sepsis requiring fluid resuscitation (cases) and without severe sepsis (controls) and their outcome.

Characteristics	Cases (n = 50)	Controls (n = 354)	OR	95% CI	p
Male sex	25 (50)	203 (57)	0.74	0.39–1.40	0.408
Age in months (median, IQR)*	5.9 (2.4, 11.0)	10.1 (5.0, 18.0)	-	-	<0.001
Poor socio-economic condition	42 (84)	299 (85)	0.97	0.41–2.36	0.902
Lack of BCG vaccination	15 (30)	36 (10)	3.79	1.78–8.00	<0.001
Lack of DPT, OPV, Hib and Hepatitis vaccination	14 (28)	76 (22)	1.42	0.69–2.80	0.391
Non-breastfed from neonatal period	12 (24)	51 (14)	1.88	0.86–4.02	0.123
Weight for length Z score	-4.1 ± 2.3	-3.8 ± 1.3	-0.34*	-1.01–0.34	0.320
Acute Watery Diarrhea	44 (88)	260 (73)	2.77	1.09–7.47	0.039
Reluctance to feed	5 (10)	25 (7)	1.46	0.46–4.29	0.399
Drowsiness	23 (46)	39 (11)	6.88	3.43–13.83	<0.001
Thrush	11 (22)	63 (18)	1.30	0.59–2.81	0.600
Lower chest wall in-drawing	27 (54)	143 (40)	1.73	0.93–3.27	0.095
Abdominal distension	12 (24)	40 (11)	2.48	1.12–5.41	0.022
Hypoxemia	14 (28)	28 (8)	4.53	2.05–9.93	<0.001
Hypoglycemia	2 (4)	4 (1)	3.65	0.45–23.99	0.163
Bacteremia	6 (12)	33 (9)	1.35	0.48–3.64	0.604
Hypokalemia	21 (42)	118 (33)	1.45	0.76–2.76	0.294
Hyperkalemia	6 (12)	48 (14)	.87	0.31–2.27	0.935
Hyponatremia	8 (16)	53 (15)	1.08	0.44–2.56	0.983
Hypernatremia	11 (22)	21 (6)	4.47	1.86–10.64	<0.001
Metabolic acidosis	35 (70)	141 (40)	3.52	1.78–7.04	<0.001
Acute kidney injury	20 (40)	25 (7)	8.77	4.13–18.16	<0.001
Hypocalcemia	24 (48)	75 (21)	3.43	1.79–6.60	<0.001
Hypomagnesemia	1 (2)	11 (3)	0.64	0.03–4.93	1.00
Outcome (Died)	20 (40)	15 (4)	15.07	6.57–34.86	<0.001

Figures represent n (%), unless specified. OR: odds ratio. CI: confidence interval. IQR: inter-quartile range.

*Comparison of age (median, IQR) among the groups has been done by using Mann-Whitney test in the SPSS (version 17.0) that does only provide the information of p value but no information of OR or MD (and their CIs) were available

**Table 2:** Results of logistic regression exploring independent risk factors for severe sepsis in under-five SAM children with pneumonia required fluid resuscitation.

Characteristic	OR	95% CI	p
Young infancy (median: 5.9 months; IQR: 2.4–11 months)	1.04	0.98–1.08	0.060
Lack of intake of BCG vaccination	4.60	1.99–10.66	<0.001
AWD	1.22	0.40–3.72	0.726
Drowsiness	3.39	1.52–7.57	0.003
Abdominal distension	2.94	1.25–6.92	0.014
Hypoxemia	1.20	0.42–3.42	0.729
Hypernatremia	1.27	0.45–3.59	0.656
Metabolic acidosis	2.21	1.01–4.87	0.050
Acute kidney injury	3.24	1.36–7.74	0.008
Hypocalcemia	2.03	0.97–4.22	0.060

## Results

In total, 1,482 SAM children were admitted to the Dhaka Hospital of icddr,b during the study period, and 404 of them fulfilled our inclusion criteria of whom 50 (12%) were cases and 354 were controls. Out of the 50 cases, 29 (58%) did not respond to fluid resuscitation, required inotrope (s) support, and regarded as septic shock; 29 of the total 404 study children (7%) had septic shock. The case-fatality rate in children with severe sepsis was 40% (20/50) and that among children with septic shock was 69% (20/29).

The case-fatality-rate was significantly higher among the cases than the controls (Table 1). The cases more often were younger, and had AWD, hypoxemia, hypernatremia and hypocalcemia compared to the controls (Table 1). During hospitalization, 14 (28%) cases and 28 (8%) controls required bubble CPAP oxygen therapy, 29 (58%) cases and no control received inotropes, and 25 (50%) cases and 19 (5%) controls had mechanical ventilation. In logistic regression analysis, after adjusting for potential confounders the cases were independently associated with lack of BCG vaccination, and drowsiness, abdominal distension, acute kidney injury, and metabolic acidosis at admission (Table 2). There was no evidence of differences in distributions of sex; poor socio-economic condition; lack of DPT, OPV, Hib and Hepatitis vaccination; lack of breast feeding since neonatal period; weight for length Z score; reluctance to feed; presence of thrush and lower chest wall in-drawing, hypoglycemia, bacteremia, hypokalemia, hyperkalemia, hyponatremia and hypomagnesemia at admission; and development of hospital acquired infection among the cases and the controls (Table 1).

## Discussion

The observation of significantly higher case-fatality in pneumonic SAM children with severe sepsis compared to those without severe sepsis is an important but understandable observation. Data from a number of recent studies conducted in children in the critical care ward are consistent with our observation [14, 26, 27]. Severe malnutrition is associated with depressed cell mediated and humoral immune responses [28, 29], and is highly susceptible to infectious disease, including pneumonia [18], and is often associated with severe sepsis [30]. Pneumonia in SAM children having severe sepsis is also associated with oxidative stress that leads to endogenous production of nitric oxide [31]. This nitric oxide is responsible for uncontrolled vasodilatation and hypotension leading to death [32, 33]. On the other hand, both high intracellular and high extracellular sodium content and simultaneously reduction of alveolar epithelial sodium and chloride transport in severely malnourished children impedes clearance of fluid from the alveolus [34, 35]. This event may be more pronounced in SAM children having pneumonia and severe sepsis. These children undergoing fluid resuscitation may contribute to the development of interstitial edema, yet may not exhibit overt clinical sign, but still culminate in death [22, 31]. Our study children with severe sepsis received fluid resuscitation following our hospital guideline that is based on robust hospital data from Bangladesh [18]. Results of study conducted in Africa (FEAST trial) observed higher deaths in association with aggressive fluid therapy in children with features of severe sepsis [17]. The FEAST trial did not include SAM children and did not receive any non-invasive or invasive respiratory support for those who developed respiratory failure, whereas all of our study population were SAM children and they had the opportunity to receive bubble CPAP or mechanical ventilation following standard hospital guideline based on local evidence [20]. Thus, compared to the children in FEAST trial, our study children were sicker. However, the possible impact of WHO recommended relatively slower and less aggressive fluid resuscitation on case fatality in our study population remains unknown. Echocardiographic findings in severely malnourished children are variable but include reduced left ventricular mass [36, 37] and reduction in left ventricular ejection fraction in association with severe infection [36, 38]. Thus, the abnormal cardiac activity might have contributed to deaths in our study children with severe sepsis. However, our hospital doesn’t have the facility and we could not perform echocardiography, which could be done in follow up, randomized, controlled clinical trial to evaluate the efficacy of different fluid resuscitation strategies in managing severe sepsis in pneumonic SAM children including evaluation of cardiac mass and function by echocardiography. There is no published data from randomized controlled clinical trial on fluid resuscitation, with or without involving inotropes in SAM children with severe sepsis, although the burden of severe sepsis in SAM children is perceived to be high! Thus, more research is imperative to have an evidence base robust management algorithm in such children. Otherwise, earlier referral to critical care ward will not be sufficient enough to change the outcome in such children.

The observation of association of drowsiness on admission, abdominal distension, acute kidney injury, and metabolic acidosis with severe sepsis in pneumonic SAM children who received fluid resuscitation were not surprising findings. Drowsiness as an independent predictor of severe sepsis in under-five children is consistent with the findings of a recent study from Bangladesh [14]. Children with severe sepsis often experience poor splanchnic circulation, while severe infection such as severe sepsis contributes to small bowel overgrowth in severely malnourished children [31]. The combination of poor splanchnic circulation, small bowel overgrowth, and hypokalemia may contribute to abdominal distension, although in this study the distribution of hypokalemia was similar among the cases and the controls. Severe sepsis is also associated with disordered microcirculation and increased lactate production as a by-product of anaerobic cellular respiration leading to metabolic acidosis as noted in our study population [39]. This finding is also consistent with a recently published study from Bangladesh [40]. Impaired hemodynamic and hypotension, recognised features of severe sepsis, may lead to an under-perfusion of kidneys and acute kidney injury in severely malnourished children. Additionally, tubular cell apoptosis and microvascular thrombosis, both driven by systemic release of proinflammatory cytokines and features of severe sepsis, are important contributors to acute kidney injury in such children [41].

Our observation of the association of lack of BCG vaccination with severe sepsis in severely malnourished pneumonic children is a novel finding, although the beneficial effects of BCG vaccination on non-tubercular illness has been well documented [42]. Lack of BCG usually is a marker of lack of uptake of services that might be due to a lack of adequate knowledge of caregivers about the well known tubercular and non-tubercular advantages of BCG vaccination. BCG vaccination has been reported to reduce around 50% of deaths from non-tubercular infections such as pneumonia in developing countries with high childhood mortality [43]. This may reflect growing evidence on substantial heterologous effects of BCG in children [42] including its potentials for reducing the incidence of severe sepsis in TB endemic countries. This is important information for the clinicians as well as policymakers, especially in developing countries with high burden of tuberculosis and childhood deaths for promoting BCG vaccination.

The retrospective nature of the data limited systematic collection of data, which is an important limitation of our study.

Based on results of our study and within the afore-mentioned limitations, we may suggest that pneumonic SAM children with severe sepsis requiring fluid resuscitation in addition to standard antibiotic and other supportive therapy are likely to have higher case-fatality compared to those without severe sepsis. Our study also identified several simple independent predicting factors for severe sepsis those may help clinicians to initiate early intervention in pneumonic SAM children especially in resource poor settings. The results highlight the importance of randomized, controlled clinical trials in evaluating the efficacy of different fluid resuscitation strategies with or without inotropes support in order to reduce deaths in such children.
